# Enhancement of osteogenic differentiation of rat adipose tissue-derived mesenchymal stem cells by zinc sulphate under electromagnetic field via the PKA, ERK1/2 and Wnt/β-catenin signaling pathways

**DOI:** 10.1371/journal.pone.0173877

**Published:** 2017-03-24

**Authors:** Ezzatollah Fathi, Raheleh Farahzadi

**Affiliations:** 1 Department of Clinical Sciences, Faculty of Veterinary Medicine, University of Tabriz, Tabriz, Iran; 2 Cardiovascular Research Center, Tabriz University of Medical Sciences, Tabriz, Iran; Van Andel Institute, UNITED STATES

## Abstract

Zinc ion as an essential trace element and electromagnetic fields (EMFs) has been reported to be involved in the regulation of bone metabolism. The aim of this study was to elucidate the effects of zinc sulphate (ZnSO_4_) on the osteogenic differentiation of adipose tissue-derived mesenchymal stem cells (ADSCs) in the presence of EMF as a strategy in osteoporosis therapy. Alkaline phophatase (ALP) activity measurement, calcium assay and expression of several osteoblastic marker genes were examined to assess the effect of ZnSO_4_ on the osteogenic differentiation of ADSCs under EMF. The expression of cAMP and PKA was evaluated by ELISA. The expression of β-catenin, Wnt1, Wnt3a, low-density lipoprotein receptor-related protein 5 (LRP5) and reduced dickkopf1 (DKK1) genes were used to detect the Wnt/β-catenin pathway. It was found that ZnSO_4_, in the presence of EMF, resulted in an increase in the expression of osteogenic genes, ALP activity and calcium levels. EMF, in the presence of ZnSO_4_, increased the cAMP level and protein kinase A (PKA) activity. Treatment of ADSCs with (MAPK)/ERK kinase 1/2 inhibitor, or PKA inhibitor, significantly inhibited the promotion of osteogenic markers, indicating that the induction of osteogenesis was dependent on the ERK and PKA signaling pathways. Real-time PCR analysis showed that ZnSO_4_, in the presence of EMF, increased the mRNA expressions of β-catenin, Wnt1, Wnt3a, LRP5 and DKK1. In this study, it was shown that 0.432 μg/ml ZnSO_4_, in the presence of 50 Hz, 20 mT EMF, induced the osteogenic differentiation of ADSCs via PKA, ERK1/2 and Wnt/β-catenin signaling pathways.

## Introduction

Osteoporosis is the most common metabolic bone disorder that frequently afflicts many women after menopause and some men in older age. It is characterized by low bone mass and structural deterioration of bone tissue, resulting in pain, physical disability, high cost of therapy and increased susceptibility to fractures [1]. All fractures (wrist, ribs, vertebrae, and hip) are associated with considerable morbidity and a decline in quality of life [2]. Of all fractures due to osteoporosis, hip fractures are the most disabling [3]. A great deal of evidence suggests that osteoporosis results from the interaction of genetic susceptibility and environmental factors [4]. Among which, zinc ion (Zn^+2^) as an essential trace element, was reported to be involved in the pathogenesis of osteoporosis and has direct effect on bone mineralization [5–8]. It has been reported that the administration of zinc sulphate (ZnSO_4_) to weanling rats for 3 days resulted in a statistically significant increase in bone development [9]. This increase in bone formation is accompanied by other biochemical changes associated with bone growth such as elevated alkaline phosphatase levels and a statistically significant increase in bone zinc levels [10]. It was reported that zinc could also inhibit pit formation by isolated neonatal rat osteoclasts in a biphasic manner (10^−14^–10^−10^ mol/L and 10^−4^ mol/L), but had no effect on osteoclast number [11]. In addition, it helps in the synthesis of protein in osteoblastic cells and plays a role in the preservation of bone mass [12]. It is believed that eluted Zn^+2^ supplementation stimulated osteoblast and osteoblast differentiated gene expression [7]. Yusa et al. (2011) reported that Zn^2+^ releasing titanium implants result in the stimulation of cell viability, osteoblastic marker gene expression such as type I collagen, osteocalcin (OCN), alkaline phosphatase (ALP), and calcium deposition in human bone marrow-derived mesenchymal stem cells [7]. In addition to the use of Zn^2+^ in promoting bone formation; the use of electromagnetic fields (EMFs) exposure is another method in the treatment of osteoporosis. The therapeutic effects of EMFs on bone were indicated by Bassett et al. (1974), whose reports led to some clinical trials and widespread commercial availability [13]. The practical clinical use of EMFs in the treatment of osteoporosis has been restricted due to parameters such as the uncertainty of frequencies, intensities, duty ratio and duration of exposure [14]. Tsai et al. (2009) showed that the production of ALP and expression of osteogenic genes, including Runt-related transcription factor 2 (Runx2) and ALP, were stimulated in human mesenchymal stem cells (hMSCs) by pulsed electromagnetic field (PEMF) exposure [15]. Manjhi et al. (2013) reported that chronic (2 h/d × 8 wk) EMF exposure (17.96 μT, 50 Hz) to spinal cord injury (SCI) in rats is effective in attenuating SCI-induced osteoporosis [16]. In a study by Lim et al. (2013), it was demonstrated that ELF-PEMF significantly cause the alteration of osteogenesis-related genes expression in human alveolar bone-derived mesenchymal stem cells [17]. Yan et al. (2015) indicated that 0.6 mT, 50 Hz PEMF stimulates both proliferation and osteogenic differentiation of rat calvarial osteoblasts [14]. In other studies, it was shown that EMF stimulation accelerated the up- or down-regulation of some genes [18, 19]. Although, these changes in gene expression could partially explain the mechanism of osteogenesis promotion by EMFs, the detailed mechanism of EMF effects is still unknown. Zhou et al. (2012) indicated that PEMFs had positive effects on bone mass and prevents the deterioration of bone microarchitecture and strength in ovariectomy rats [20]. At this time, many researchers have tried to investigate the mechanism of physical–chemical interactions between biological tissues and EMF. Some researchers generally believed that changes in gene and protein expression have often coincided with the activation or suppression of the signaling pathway [14, 21]. Therefore, studies are required to fully elucidate the complexity and diversity of the signaling pathways involved in diseases. The canonical Wnt signaling plays a role as key regulator in the osteogenic differentiation of mesenchymal progenitor cells, bone formation and various aspects of skeletal development [22]. Other signaling pathways implicated in the osteogenic differentiation are the MAPK/ERK and cAMP/PKA pathways that have been reported to regulate the proliferation and differentiation of bone cells during osteogenic differentiation [23, 24]. Li et al. (2006) reported that PEMF exposure led to an increase in cytosolic Ca^2+^ and activation of calmodulin, which is also the mechanism of the osteogenesis process [25]. [26]. Hou et al. (2006) indicated that EMF activated the extracellular signal-regulated kinase mitogen-activated protein kinase (ERK–MAPK) and p38 pathways [27]. In a study by Yong et al. (2014), it was demonstrated that 15 Hz, 1 mT promoted mesenchymal stem cells (MSCs) osteogenesis and that the EMF-induced osteogenic markers were mediated by both the PKA and MAPK signaling pathways [28]. In fact, EMFs activate some of the signaling pathways.

The use of human beings as the test model is impossible and the long-term observation of a test group could be affected by other factors, such as dietetic habit, inherited disease etc. Hence, by creating an amplified EMF, one can imitate these long-term effects in a relatively short time [29]. Certainly, stem cells could be the best choice. A slight influence on the stem cells may cause unexpected consequences to its related adult cells [30]. MSCs are pluripotent cells and are characterized by their self-renewal abilities through mitotic cell division, as well as by their potential to differentiate into multiple types of lineages such as: chondrogenic, osteogenic, adipogenic, myogenic, neurogenic, and hepatogenic [31, 32]. Adipose tissue has become a rich source of MSCs, providing abundant and accessible source of ADSCs with minimal patient discomfort. Although, it lacks donor limitation, obtaining these stem cells may pose a low risk of side effects [33].

The aim of this study was to evaluate the effects of 50 Hz, 20 mT EMF in the presence of 0.432 μg/ml ZnSO_4_, on the osteogenic differentiation of ADSCs in order to further elucidate the simultaneous effects of ZnSO_4_ and EMF on the pathogenesis of osteoporosis. The first question was whether EMF in the presence of ZnSO_4_ could promote osteogenic differentiation of ADSCs and osteogenic gene expression. The second question, related to ERK-MAPK, cAMP–PKA and Wnt/β-catenin pathways, was whether these pathways could be activated by EMF in the presence of ZnSO_4_.

## Materials and methods

### Reagents

All materials were purchased from Sigma-Aldrich (St. Louis, MO, USA), unless otherwise stated. All tissue culture plastic ware was from SPL Life Sciences (Pocheon, Korea). All experimental procedures were repeated for three times. About 5 (6- to 8-week-old) male Rattus rats were purchased from Drug Applied Research Center, Tabriz University of Medical Sciences (Tabriz, Iran), and euthanized using ketamine (87 mg/kg)/ Xylazine (13 mg/kg).

### Isolation, culture and osteogenic induction of ADSCs

In this research, for experimentation, consent was given by the ethical committee of the Faculty of Veterinary Medicine, University of Tabriz (Tabriz, Iran) according to the guidelines of the Helsinki ethical code regarding experiments performed on animals. Epididymal adipose tissue was obtained from 5 male Rattus rats with 6–8 weeks age under sterile conditions and placed in a sterilized culture dish under a laminar hood. Fat tissue was carefully dissected and minced using a sterile scissors. The fat was washed extensively with Dulbecco’s modified Eagle’s medium (DMEM, Gibco, UK) supplemented with 10% (v/v) fetal bovine serum (FBS) and 5% penicillin/streptomycin. Tissue was then enzymatically dissociated for 30 min at 37°C using 0.075% (w/v) collagenase type I (Invitrogen, UK). The solution neutralized by the addition of DMEM containing 10% (v/v) FBS, was passed through a 70-μm filter to remove undissociated tissue and centrifuged at 800×g (2610 rpm) for 5 min. The cell pellet was resuspended in DMEM containing 10% (v/v) FBS and 1% (v/v) penicillin/streptomycin solution [34, 35]. Cultures were maintained at subconfluent levels in a 37°C incubator with 5% CO_2_ and passaged with 0.25% trypsin (Gibco, UK) and 1 mM ethylenediaminetetraacetic acid (EDTA; Invitrogen, UK) when required. Osteogenic differentiation was induced with osteogenic differentiation medium containing 10% FBS, 10 nM dexamethasone, 10 mM b-glycerophosphate, and 0.05 mM L-ascorbic acid-2-phosphate for 21 days. The medium was replaced with fresh osteogenic differentiation medium every 3–4 days during the 21 days of cultivation.

### ELF magnetic field and exposure system

The EMF device was designed and manufactured by the University of Tabriz in Iran. EMF was generated by a parallel set of Helmholtz solenoid coils with 500 turns of 0.7 mm coated copper wire. Each solenoid diameter was 27 cm. The coil was placed into the cell incubator; the field was set to 50 Hz electromagnetic field frequency and generated a magnetic flux density of 20 mT as previously described by Yan et al. (2010). The EMF exposed plates were placed in the center half way between the plains of coils to receive a uniform field for 30 min per day for 21 days. Sham-exposed control samples were kept under the same conditions but in another incubator, without using EMF. The CO_2_ concentration, temperature and humidity of the sham-exposed control samples were similar. The value of the alternating magnetic field was measured continuously using an EFA-2 field analyzer with a 3 cm diameter probe [29].

### MTT assay for cell proliferation activity of ADSCs in the presence of ZnSO_4_

The MTT (3-(4, 5-dimethylthiazol-2-yl)-2, 5-diphenyl tetrazolium bromide) test measures the mitochondrial activity in the cell culture, which reflects the number of viable cells. In brief, cells (at passage 3–6) were trypsinized and seeded at a density of 2 × 10^3^ cells per well to a 96-well culture plate. ZnSO_4_ was added to the wells at final concentrations of 4.32, 0.432, 0.0432 and 0.00432 μg/ml. Control wells were prepared by addition of corresponding medium. The stock MTT dye solution (5 mg/mL) was added to each well after the plates were incubated at 37°C in a 5% CO_2_ incubator for 21 days. Following incubation for 4 h, the supernatant was removed and 100 μL of dimethyl sulfoxide was added to each well. The optical density of each well was measured in an ELISA Reader (Labsystems, Helsinki, Finland) at a wavelength of 570 nm [5, 8].

### Phenotypical characterization of ADSCs

#### Detection of ADSCs markers by flow cytometry

To analyze cell surface markers, Approximately 10 × 10^5^ ADSCs from the passage 4 cultures were harvested and incubated with an appropriate amount of fluorescein isothiocyanate (FITC) or FITC-conjugated mouse primary CD44, CD90, CD105, CD31, CD34 and CD56 antibodies (BD Phar-mingen, San Diego, CA, USA) (1μg/10^6^cells) in phosphate-buffered saline (PBS) (supplemented with 1% bovine serum albumin) for 30 min on ice. Fluorescence activated cell sorter (FACS) analysis was also done using a FACScan (Becton Dickinson Franklin Lakes, USA) and data were analyzed with a FlowJo software (version 6.2).

#### Osteogenic differentiation of ADSCs

To promote osteogenic differentiation, cells at passage 4 were seeded at an initial density of 50 × 10^3^ cells in each well of 6-well plates and treated with osteogenic induction medium as mentioned above. After 21 days of culture, cells were washed with PBS and fixed in a solution of 2% (v/v) formaldehyde. After 15–20 min, alizarin red 40 mM (pH 4.1) was added to each well. The plates were incubated at room temperature for 20 min with shaken and then they were washed 2–3 times with PBS shaken for 5 min to reduce nonspecific staining [5, 36].

#### Adipogenic differentiation of ADSCs

To promote adipogenesis, subconfluent cells were incubated in adipogenic medium containing 0.5 mM 1-methyl-3 isobutylxanthine, 1μM dexamethasone, 10 μg/ml insulin, and 200 μM indomethacin; the medium was changed every 3–4 days. At the end of the day 21, formalin-fixed cells were washed in 50% isopropanol and stained with oil Red for 15 min and observed by a light microscope [17].

#### Chondrogenic differentiation of ADSCs

rADSCs were seeded the same as adipogenic and osteogenic differentiation. Chondrogenic differentiation medium composed of high-glucose DMEM with 10% FBS, 100 U/mL penicillin, 100 μg/mL streptomycin, 1.3 mM L-ascorbic 2-phosphate, 0.01 mM dexamethasone and ITS+1 Liquid Media Supplement (100x) was used for 14 days. At the end of chondrogenesis, cells were fixed with 4% (v/v) paraformaldehyde for 30 min, washed with PBS for three times and proteoglycans were stained with 0.1% toluidine blue [36].

#### Neurogenic differentiation of ADSCs

To induce neuronal differentiation, subconfluent cells were seeded in 6-well plates at 20 × 10^3^ cells/cm2 containing complete culture medium for 24 h. The medium was then changed into neurogenic medium containing high glucose DMEM supplemented with 10% FBS, 20 ng/ml bFGF, 20 ng/ml EGF, 10 ng/ml GDNF and 200 ng/ml L-ascorbic acid-2-phosphates for 14 days. The medium was changed with fresh neurogenic differentiation medium every 3–4 days. Neural differentiation was assessed by Nissl bodies staining with 0.5% cresyl violet [37].

### ALP activity assay

The ADSCs (from passage 4) were plated at a density of 10 × 10^4^ cells/well in 24-well plates containing an osteogenic induction medium for 21 days, and were divided into four groups: group I (control without any ZnSO_4_ treatment and EMF exposed), group II (50 Hz, 20 mT EMF exposed without ZnSO_4_ treatment), group III (0.432 μg/ml ZnSO_4_ treatment without EMF exposed) and group IV (50 Hz, 20 mT EMF exposed with 0.432 μg/ml ZnSO_4_ treatment). The exposure time duration for EMF exposed groups were 30 min per day for 21 days. Every 4 days, cultured cells were washed twice with PBS and incubated with a cell lysis buffer (1.5 M Tris (hydroxymethyl) aminomethane, 1 mM ZnCl_2_, 1mM MgCl_2_, 1%Triton-X100, pH 9.2) by shaking at 37°C for 30 min. Subsequently, 250 μl of the substrate solution was added to each well and incubated at 37°C for 15 min. The ALP enzyme activity of the cell was determined by measuring the conversion of p-nitrophenyl phosphate to p-nitrophenol. The substrate solution was prepared by dissolving 4-nitrophenyl phosphate disodium salt hexahydrate into a substrate buffer consisting of 50 mmol/L glycine and 1 mmol/L MgCl_2_ at a pH of 10.5. ALP activity was calculated after measuring the absorbance of p-nitrophenol product formed at 405 nm on an ELISA microplate reader.

### Calcium assay

The ADSCs (from passage 4) were plated at a density of 10 × 10^4^ cells/well in 24-well plates containing an osteogenic induction medium for 21 days, and were divided into four groups as described in section 2.6. The exposure time duration for EMF exposed groups were 30 min per day for 21 days. Every 4 days, cultured cells were rinsed twice with PBS and trypsinized with Trypsin-EDTA. Subsequently, the suspension was collected to the 2 ml tube and treated with ultrasonication, and the supernatant was used for calcium determination according to the manufacturer’s instruction contained in calcium assay kit (Sigma, USA). Absorbance of samples was read at 570 nm after the addition of reagents. The total calcium was calculated from standard solutions prepared in parallel, and expressed as μg/well [29].

### cAMP assay

Intracellular cAMP assay was performed using the cAMP Enzyme Immunoassay Kit (Sigma, USA), according to the manufacturer’s guidelines. Briefly, the ADSCs (from passage 4) were plated at a density of 10 × 10^4^ cells/well in 24-well plates containing an osteogenic induction medium for 21 days, and were divided into four groups as described in section 2.6. The exposure time duration for EMF exposed groups were 30 min per day for 21 days. After the termination of EMF exposure at the end of the 21st day, the plates were kept in the incubation chamber for 5, 15, 30, 45, 60, 90 and 120 min before cells were collected for cAMP analysis [28, 38]. The cells were rinsed twice with PBS, trypsinized with Trypsin-EDTA and lysed with lysis buffer. The cAMP level was then measured using the cAMP Enzyme Immunoassay Kit, a Non-radioactive, competitive enzyme immunoassay method.

### PKA catalytic activity assay

The ADSCs were plated in the same manner as described for the intracellular cAMP assay. After termination of EMF exposure at the end of the 21st day, the plates were kept in the incubation chamber for 30, 60, 90 and 120 min before cells were collected for PKA activity assay. PKA activity was assayed in cell lysates using the Pep Tag assay for non-radioactive detection of PKA (Promega Corperation, Madison, WI, USA), on the basis of the phosphorylation of fluorescent-tagged PKA-specific peptides. The cell lysate for PKA protein was prepared using a cold PKA extraction buffer (25 mM Tris-HCl, pH 7.4, 0.5 mM EDTA, 0.5 mM EGTA, 10 mM β-mercaptoethanol, 1 μg/ml leupeptin and 1 μg/ml aprotinin). Aliquots of the PKA preparation and PKA catalytic subunit as a positive control were incubated for 30 min at 30°C in PepTag PKA 5x reaction buffer (100 mM Tris–HCl, pH 7.4, 50 mM MgCl2, and 5 mM ATP) and 0.4 μg/μl of the PKA-specific peptide substrate PepTag A1 (L–R–R–A–S–L–G; Kemptide). The reaction was stopped by heating for 10 min at 95°C. Phosphorylated and unphosphorylated PepTag peptides were separated on a 0.8% agarose gel by electrophoresis. Phosphorylated peptide migrated toward the cathode (+), while nonphosphorylated peptide migrated toward the anode (–).

### RNA extraction and cDNA synthesis

Cells were cultured at a concentration of 40 × 10^4^ cells/well in 6-well plates containing an osteogenic induction medium for 21 days, and were divided into four groups as described in section 2.6. The exposure time duration for EMF exposed groups were 30 min per day for 21 days. After termination of EMF exposure at the end of the 21st day, total RNA from the cells was isolated using TRIzol reagent (Invitrogen Life Technologies, Burlington, Canada). Extracted cellular RNA was dissolved in DEPC-treated water. cDNA synthesis was carried out using the RevertAid ^TM^ first strand cDNA synthesis kit (K1622; Fermentas, Germany). 2 μg RNA was used for the first strand cDNA synthesis in a total volume of 20 μL according to the manufacturer’s guidelines. For every reaction set, one RNA sample was prepared without RevertAid ^TM^ M-MuLV reverse transcriptase (RT reaction) to provide a negative control in the subsequent PCR.

### Quantitative Real-time PCR measurements of gene expression

All PCR reactions were performed using the Corbett Rotor-Gene™ 6000 HRM (Corbett Research, Australia) in a total volume of 20 μL containing Power SYBR Green master mix (2x) (TaKaRa Ex Taq HS, Japan), Primer fwd (0.5 μM), Primer rev (0.5 μM), cDNA (30 ng/μl) and H_2_O. The mRNA expressions of target genes in the ADSCs included ALP, OCN, bone morphogenetic protein-2 (BMP2), Runx2, β-catenin, Wnt1, Wnt3a, low-density lipoprotein receptor-related protein 5 (LRP5), and dickkopf1 (DKK1). PCR amplifications were done in glass capillary tubes. β-actin was selected as an endogenous housekeeping gene. The thermal cycling conditions were beginning denaturation step 5 min at 95°C, followed by 40 cycles, each denaturation at 95°C for 10 s, annealing at 60°C (ALP, β-catenin and Wnt1) or 61°C (OCN, Wnt3a, LRP5) or 59°C (Runx2, BMP2 and DKK1) for 15 s and extension at 72°C for 20 s. Fluorescence data was analysed by using Rotor-Gene 6000 Software version to get CT values. The CT values were calculated in relation to β-actin CT values by the 2^-ΔΔCT^ method, in which ΔCt was the difference between the Ct value of genes and the Ct value of β-actin [39]. Primers were designed using Oligo 7 v.7.52 software (Molecular Biology Insights, Inc, USA) and the sequences for each primer are presented in Table 1.

**Table 1:** Primer sequences used for the real-time PCR assays.

No.	Gene	Primer pair sequence (5'-3')	Product length (bp)
NM_013059.1	ALP	CCTTGAAAAATGCCCTGAAA CTTGGAGAGAGCCACAAAGG	191
NM_001270638.1	OCN	GTGACAGTATTAGCGAGTGGG ATTGCGAGTTCCAGTGCC	201
NM_017178.1	BMP2	CCATCACGAAGAAGCCATCGAG CTTCCTGCATTTGTTCCCGAA	143
NM_001278484.2	Runx2	AGTAGCAAACCGAAACAC GAAATAGGCATCAGACAAA	174
NM_053357.2	β-catenin	CTGTTCTACGCCATCACC TTTCCTGATTGCCGTAAGC	178
NM_001105714.1	Wnt1	GGGGAGCAACCAAAGTCG TGGAGGAGGCTATGTTCACG	187
NM_001107005.2	Wnt3a	ATGTTCGGGACCTATTCCA CTGTAGCATCTCGCTTCC	123
NM_001106321	LRP5	GACATTTACTGGCCCAATGG CTGCCCTCCA CCACCTTCT	131
NM_001106350	DKK1	TTTCCCTAAGTGACCGACAG TGGGACCATTCTTCAGCA	158
NM_017008.4	GAPDH	AACGACCCCTTCATTGACC TCCACGACATACTCAGCACC	191

### Statistical analysis

The results were analyzed using the software program Graph Pad Prism version 6.01. We used one-way and two-way ANOVA followed by Dunnett’s post hoc test to determine the significant difference among groups. Statistical significance was determined at p<0.05. All experimental procedures were repeated for three times.

## Results

### Characterization of ADSCs

**Fig 1:**
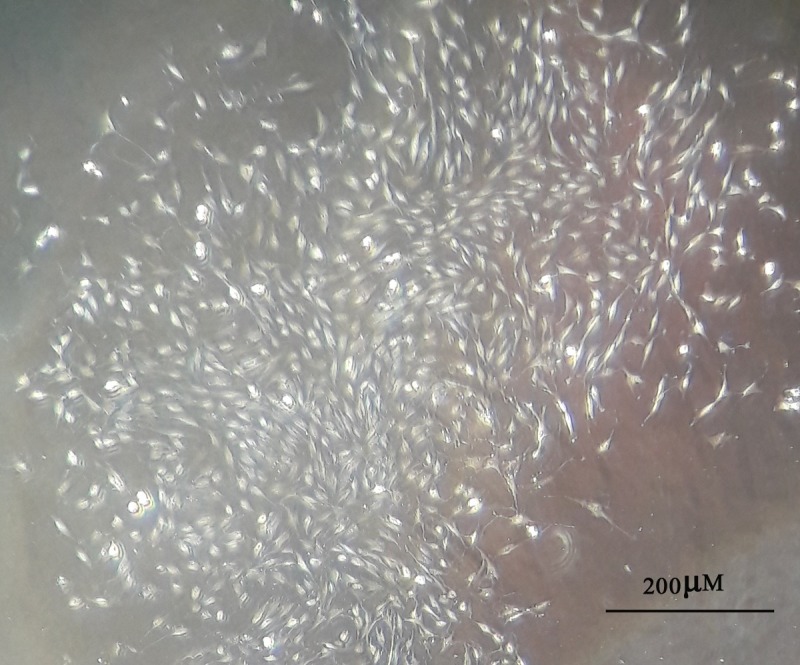
Spindle-shaped morphology of rat Adipost tissue-MSCs 7 day after seeding (bar = 200μm).

**Fig 2:**
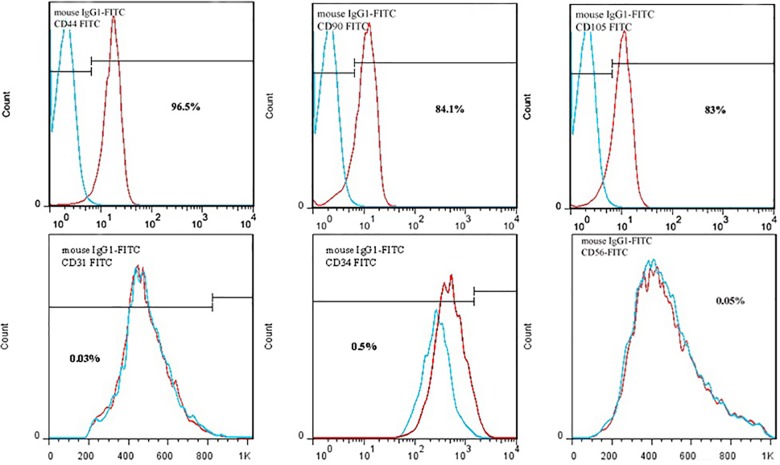
Flow cytomertic analysis of rat Adipost tissue-MSCs (rADSCs). rADSCs are stained with monoclonal antibodies directed against either CD44, CD90, CD105, CD31, CD34 and CD56 and coupled to fluorescein isothiocyanate (FITC); these signals are indicated as red lines, An isotype matched monoclonal antibody served as a control; these signals are indicated as dotted lines. rADSCs were positive for CD44, CD90 and CD105 and negative for CD31, CD34 and CD56.

ADSCs had the capacity to adhere to culture plastic flasks, and morphologically they appeared as spindle-shaped cells both as scattered individuals and in small colonies (Fig 1). Flow cytometric analysis showed that ADSCs had high levels of expression of CD44 (96.5%) CD90 (84.1%) and CD 105 (83%), and hematopoietic cell lineage-specific antigens, such as CD31 (0.03%), CD34 (0.5%) and CD56 (0.05%) were not expressed in these cells (Fig 2).

### Multilineage differentiation of ADSCs: osteogenesis, adipogenesis, chondrogenesis and neurogenesis

**Fig 3:**
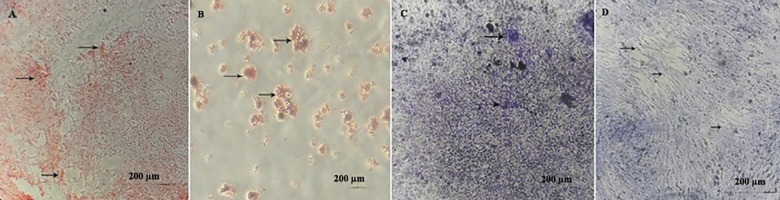
In vitro multilineage differentiation of rat Adipost tissue-MSCs (rADSCs). (A) Osteogenic differentiation and cell aggregates (were stained with alizarinred staining). Arrows show some of the mineralized cell aggregates (bar = 200μm). (B) Differentiation into adipose cells. Arrows show lipid vacuoles generated after adipose differentiation (bar = 200μm). (C) Differentiation of rADSCs to chondrocytes, proteoglycan aggregates stain with toluidine blue (bar = 200μm). (D) The neuronal cells differentiated from rADSCs show extensive somata-associated accumulations of Nissl bodies stained dark black-violet (black arrows) (bar = 200μm).

The osteogenic differentiation was evident in alizarin red staining. After mentioned staining, redness of the nodules indicated the presence of mineralized compartments as a result of the osteogenic treatment. Positive adipogenic differentiation was confirmed by oil red staining. Treated ADSCs with adipogenic differentiation medium stained lipid droplets. At the end of chondrogenesis, toluidine blue was used to staining the aggrecan aggregates as a key molecule within the cartilage matrix. Following neural induction, neuronal differentiation was confirmed with 0.5% cresyl violet. Nissl bodies appear dark black-violet with the background remaining almost colorless (Fig 3).

### Effect of ZnSO_4_ on the ADSCs proliferation

**Fig 4:**
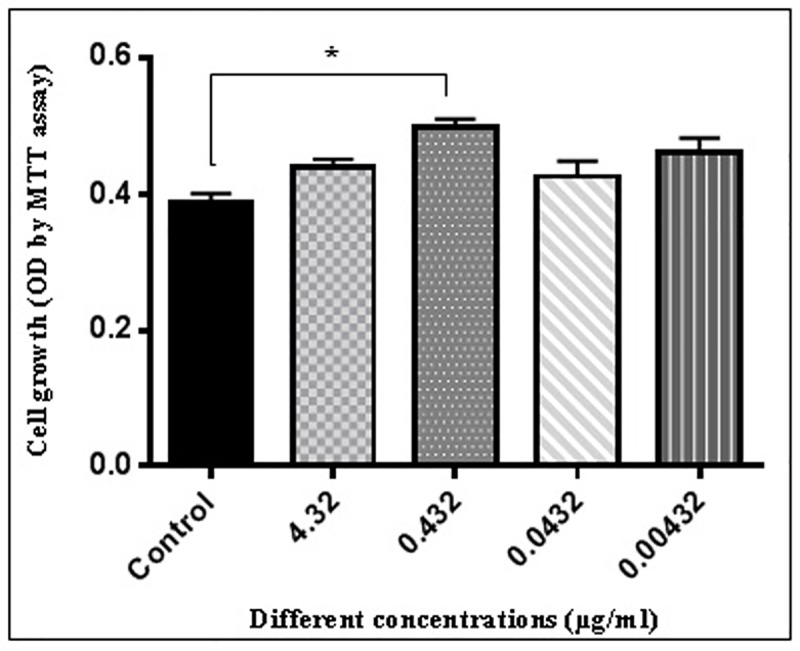
Effect of zinc sulphate (ZnSO_4_) on the proliferation of rat Adipost tissue-MSCs (rADSCs) (^*^P<0.05 vs. control, mean ± SEM; n = 3). rADSCs were cultured at the density of 2 × 10^3^ cells per well in the absence of ZnSO_4_ and in the presence of 4.32, 0.432, 0.0432 and 0.00432 μg/ml of ZnSO_4_, as described in section 2.4. MTT dye solution was added when rADSCs were cultured for 21 days. After 4 h incubation, culture medium was removed, DMSO was added and the optical density of each well was measured at a wavelength of 570 nm.

As shown in Fig 4, ZnSO_4_ had no significant effect on ADSCs proliferation at concentrations of 4.32, 0.0432 and 0.00432 μg/ml, but at concentration of 0.432 μg/ml the significant proliferation effect without cytotoxicity was seen (p<0.05). So, we used 0.432 μg/ml ZnSO_4_ in a osteogenic induction medium to treat the cells.

### The effect of ZnSO_4_ on ALP activity and calcium assay during EMF exposure

**Fig 5:**
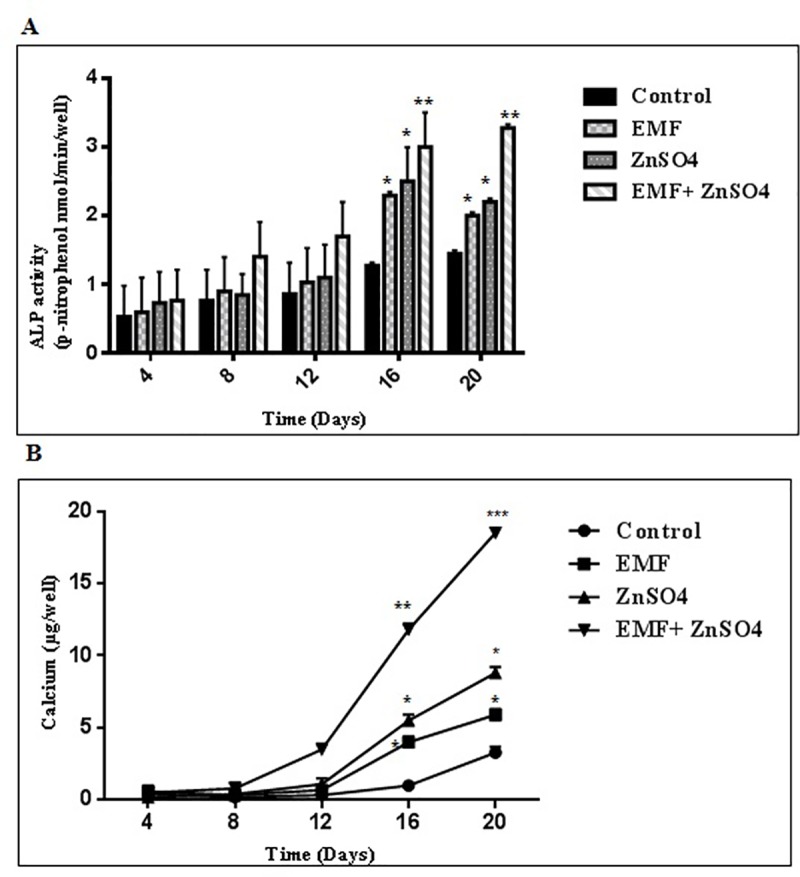
**(A) ALP activity and (B) calcium levels were affected by EMF and ZnSO**_**4**_**.** Cells were cultured in 24-well plates containing an osteogenic induction medium as described in section 2.6 and 2.7 at a density of 10 × 10^4^ cells/well. For ALP activity measurement, protein was extracted and for calcium assay, ultrasonication was done from the cultured rADSCs on days 4, 8, 16 and 20 post-EMF treatment with 50 Hz, 20 mT and post-ZnSO_4_ treatment in the concentration of 0.432 μg/ml for 30 min/day for 21 days. Likewise, protein extraction was performed on the controls; mean ± SEM; n = 3; ^*^P<0.05, ^**^P<0.01, ^***^P<0.001.

The osteogenic differentiation potential of ADSCs was quantified every 4 days. Fig 5 shows the results of ALP activity and calcium assay of ADSCs in an osteogenic medium, in the presence and absence of EMF and ZnSO_4_. A significant sharp increase in the ALP activity and calcium level was observed at days 16 and 20 of culture, respectively in group II (50 Hz, 20 mT EMF exposed without ZnSO_4_ treatment) (p <0.05), III (0.432 μg/ml ZnSO_4_ treatment without EMF exposed) (p <0.05) and IV (50 Hz, 20 mT EMF exposed with 0.432 μg/ml ZnSO_4_ treatment) (p <0.01) as compared with group I (control without any ZnSO_4_ treatment and EMF exposed). The lower and higher level of ALP activity and calcium levels were observed in groups I and IV, respectively.

### The effect of ZnSO_4_ on mRNA expressions under EMF

**Fig 6:**
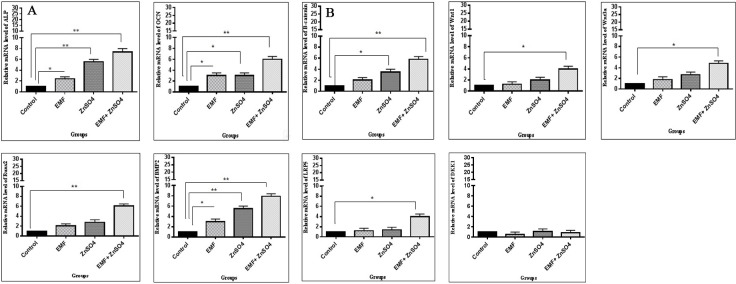
**EMF and ZnSO**_**4**_ **effect on (A) ALP, OCN, Runx2 and BMP2 and (B) β-catenin, Wnt1, Wnt3a, LRP5 and DKK1 mRNA expression in rat Adipost tissue-MSCs (rADSCs).** Cells were cultured in 6-well plates at a concentration of 30 × 10^5^ cells /well. RNA was extracted from cultured rADSCs in groups I, II, III and IV as described in section 2.10 and was subjected to Real-time PCR assay; mean ± SEM; n = 3; ^*^P<0.05, ^**^P<0.01.

To further investigate the effects of ZnSO_4_ in the presence of EMF on osteogenic differentiation and Wnt/β-catenin signaling pathway (an important pathway in bone maintenance in ADSCs), quantitative Real-time PCR was used for the detection of ALP, OCN, BMP2, Runx2, β-catenin, Wnt1, Wnt3a, LRP5 and DKK1 expression in osteoblasts, present in the osteogenic culture medium of different groups after 21 days. As shown in Fig 6, in group II (50 Hz, 20 mT EMF exposed without ZnSO_4_ treatment), the expression of ALP, OCN and BMP2 genes was significantly increased as compared with group I (control without any ZnSO_4_ treatment and EMF exposed) (p<0.05). In group III (0.432 μg/ml ZnSO_4_ treatment without EMF exposed), the expression of ALP and BMP2 genes and OCN and β-catenin genes was significantly increased as compared with group I (p<0.01 and p<0.05, respectively). In group IV (50 Hz, 20 mT EMF exposed with 0.432 μg/ml ZnSO_4_ treatment), the expression of ALP, OCN, Runx2, BMP2 and β-catenin genes and Wnt1, Wnt3a and LRP5 genes was significantly increased as compared with group I (p<0.01 and p<0.05, respectively).

### Alteration of PKA activity affected by ZnSO_4_ during EMF exposure

**Fig 7:**
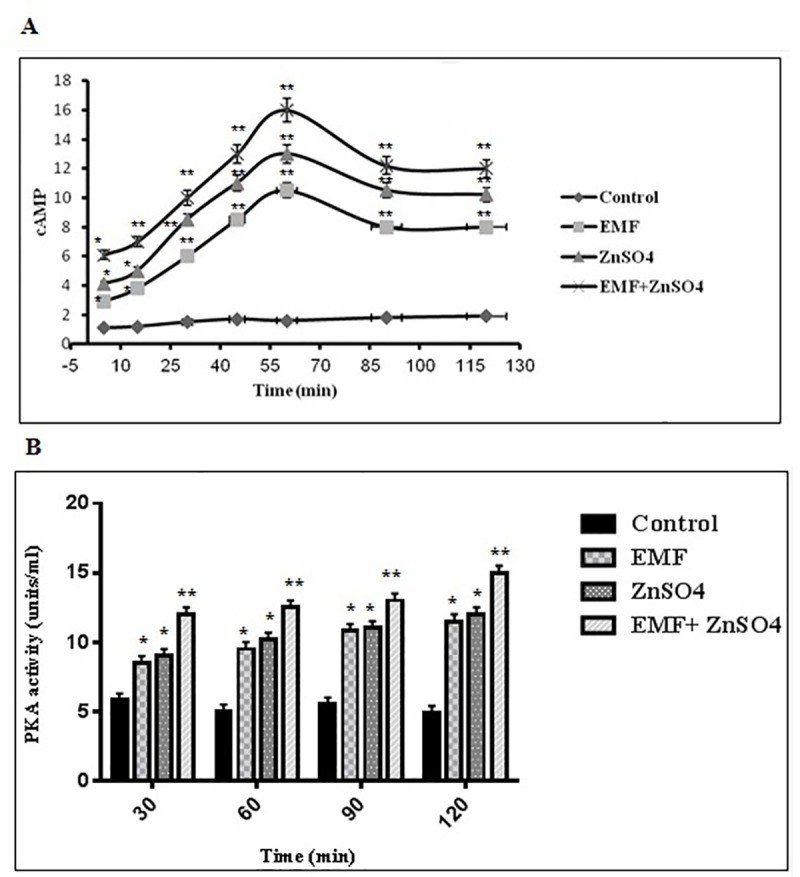
cAMP and PKA activities affected by EMF and ZnSO_4_. Cells were exposed to 50 Hz, 20 mT EMF in the presence and absence of 0.432 μg/ml ZnSO_4_ in groups I, II, III and IV as described in section 2.8 and 2.9. After the termination of EMF exposure at the end of the 21st day, the plates were kept in the incubation chamber (A) for 5, 15, 30, 45, 60, 90 and 120 min before cells were collected for cAMP analysis, and (B) for 30, 60, 90 and 120 min before PKA activity assay; mean ± SEM; n = 3; ^*^P<0.05, ^**^P<0.01.

To confirm whether cAMP–PKA was involved in the activation of osteogenic differentiation caused by ZnSO_4_ in the presence of EMF, ELISA was performed to evaluate the expression of cAMP and PKA. As shown in Fig 7A, cAMP levels were significantly increased in group II (50 Hz, 20 mT EMF exposed without ZnSO_4_ treatment), III (0.432 μg/ml ZnSO_4_ treatment without EMF exposed) and IV (50 Hz, 20 mT EMF exposed with 0.432 μg/ml ZnSO_4_ treatment) by more than 6.5, 8.1 and 10- fold, respectively at the 60 min time point, as compared with group I (control without any ZnSO_4_ treatment and EMF exposed), and decreased during 60–90 min. The cAMP level at the 120 min time point was still about 4.2, 5.3 and 6.3-fold higher in group II, III and IV, respectively than group I. The greatest increase in cAMP level was observed in group IV (p<0.01). In this study, the PKA activity was examined to further confirm the effect of ZnSO_4_ in the presence of EMF on cAMP–PKA signaling pathway. As shown in Fig 7B, the expression of PKA was significantly increased in group II, III and IV as compared with group I at 30, 60, 90 and 120 min. At 120 min, the expression of PKA was 2.3, 2.4 and 3-fold higher in group II, III and IV, respectively than group I.

### The regulation of ALP, OCN, Runx2 and BMP2 activity by PKA and ERK1/2

**Fig 8:**
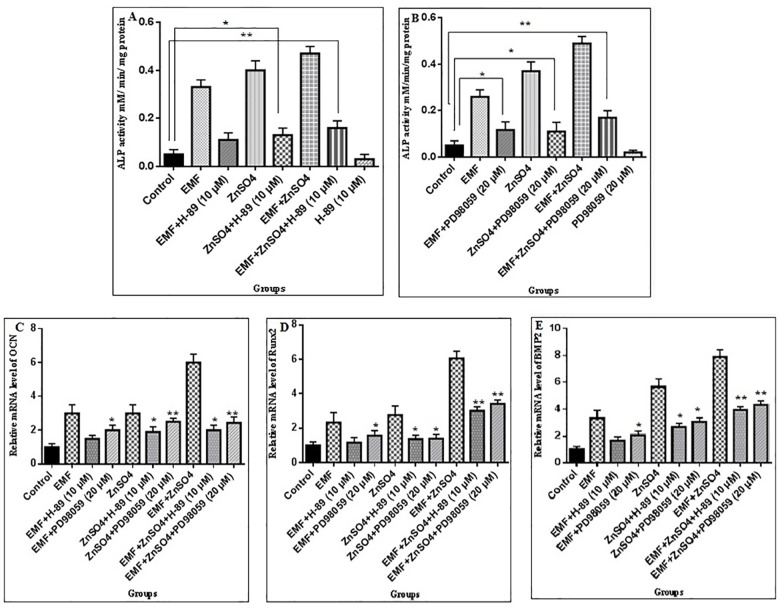
ALP, OCN, Runx2 and BMP2 are regulated by PKA and ERK1/2. The ALP activity of rat Adipost tissue-MSCs (rADSCs) treated with 50 Hz, 20 mT EMF in the presence and absence of 0.432 μg/ml ZnSO_4_ in groups I, II, III and IV as described in section 3.7 alone and plus (A) H-89 at the 10μM concentration and plus (B) PD98059 at the 20μM concentration for 6 days; Data are shown as mean ± SEM; ^*^P<0.05, ^**^P<0.01. (C) OCN, (D) Runx2 and (E) BMP2 were regulated by EMF and ZnSO_4_ requiring PKA and ERK1/2 signaling pathways. The cells were subjected to 21 days of 50 Hz, 20 mT EMF treatments in the presence and absence of 0.432 μg/ml ZnSO_4_ in groups I, II, III and IV as described in section 3.7 for 30 min/day, with 10μMH-89 or 20μMPD98059 added to the cells. RNA was extracted from cultured rADSCs in groups I, II, III and IV and was subjected to Real-time PCR assay; mean ± SEM; n = 3; ^*^P<0.05, ^**^P<0.01.

Here, we investigated whether ADSCs osteogenic differentiation promoted by 0.432 μg/ml ZnSO_4_ and 50 Hz, 20 mT EMF in group II (50 Hz, 20 mT EMF exposed without ZnSO_4_ treatment), III (0.432 μg/ml ZnSO_4_ treatment without EMF exposed) and IV (50 Hz, 20 mT EMF exposed with 0.432 μg/ml ZnSO_4_ treatment), was regulated by PKA and ERK1/2. The effects of the specific PKA inhibitor (H-89 dihydrochloride hydrate) and special ERK1/2 inhibitor (PD98059) were assessed on the ADSCs osteogenic differentiation, induced in the three aforementioned groups. H-89 was found to decrease ALP activity in group II, III and IV in a dose-dependent manner. At 10 μM, H-89 significantly decreased ALP activity as compared with the control group. PD98059 was also found to decrease ALP activity in group II, III and IV in a dose-dependent manner. At a concentration of 20 μm, ALP activity was significantly decreased, as demonstrated in Fig 8A and 8B. To investigate the effects of the PKA inhibitor (H-89 dihydrochloride hydrate) and special ERK1/2 inhibitor (PD98059) on ALP, OCN, BMP2 and Runx2, a total of 20 μm PD98059 or 10 μm H-89 were applied to the cells 1 h before treatment with 0.432 μg/ml ZnSO_4_ and 50 Hz, 20 mT EMF. The results demonstrated that the inductions of ALP, OCN, BMP2 and Runx2 by 0.432 μg/ml ZnSO_4_ and 50 Hz, 20 mT EMF in the four groups were all inhibited by PD98059 and H89, indicating that these genes were regulated by MAPK and PKA signaling pathways, as shown in Fig 8C.

## Discussion

Although the essentiality of Zn^+2^ in the preservation of bone mass and the role of various forms of EMF in osteoporosis treatment have been reported, the effect of both (Zn^+2^ and EMF) in osteoblastic bone formation and osteoporosis improvement is yet to be reported and their mechanism of action is still unknown.[7, 19, 40]. In the present study, 0.432 μg/ml was used as the final concentration of ZnSO_4_. To determine a suitable range for Zn^+2^ under this study's experimental conditions, MTT assay was used to evaluate cell viability. In this study, it was found that ZnSO_4_ had no impact on the viability of cells below 0.432 μg/ml and above 0.432 μg/ml. The exposure condition previously described by Yan et al. (2010) was used in this study. To explore the mechanism of ZnSO_4_ and EMF on osteogenic differentiation via these signaling pathways at the molecular level, we explored ALP, OCN, BMP2 and Runx2 as osteoblastic markers, as well as several important components of Wnt/β-catenin signaling pathway in response to ZnSO_4_ and EMF treatment. Thereafter, the mRNA expressions of ALP, OCN, BMP2 and Runx2 as osteoblastic markers were detected. The results of this study showed that in group II (50 Hz, 20 mT EMF exposed without ZnSO_4_ treatment), III (0.432 μg/ml ZnSO_4_ treatment without EMF exposed) and IV (50 Hz, 20 mT EMF exposed with 0.432 μg/ml ZnSO_4_ treatment), 0.432 μg/ml ZnSO_4_ and 50 Hz, 20 mT EMF promoted the mRNA expression of ALP, OCN and BMP2 as compared with group I (control without any ZnSO_4_ treatment and EMF exposed). Whereas the expression of Runx2 mRNA increased in group IV as compared with group I. The results obtained by Gaur et al. (2005) provide evidence for direct regulation of Runx2 promoter and induction of Runx2 gene expression in pluripotent mesenchymal and osteoprogenitor cells by Wnt/β-catenin signaling, and suggest that Runx2 is a target of β-catenin/TCF for the stimulation of bone formation [41]. As reported by Javed et al. (2008), Runx2 and BMP2 are important transcription factors for the promotion of osteogenic differentiation of MSCs [42]. Since OCN is a crucial protein in the expression of osteoblasts, the high expression of the OCN gene indicated that 0.432 μg/ml ZnSO_4_ and 50 Hz, 20 mT EMF could promote the osteogenic differentiation of ADSCs.

Furthermore, several studies have reported the effect of Zn^+2^ on bone formation and mineralization. Wang et al. (2007) deduced that Zn^+2^ may protect the bone by decreasing adipocytic cell formation in primary bone marrow stromal cells of mouse, which may indirectly promote osteoblast proliferation, mineralization and bone formation, and inhibit osteoclast formation, activation and bone resorption by secreting less cytosine [43]. In another investigation, the effects of adding small concentrations (2 and 5% by mole) of Zn to the sol-gel bioactive glass on the osteogenic differentiation and cell growth of MSCs were evaluated based on the ALP activity of cells. The presence of a small quantity of glass enhanced the initial cell growth, whereas ALP was significantly up regulated when grown in the presence of bioactive glass granules, particularly with larger quantity of granules and at later culture times (over 14 days) [44]. Yusa et al. (2011) reported that Zn^+2^ stimulated the viability of human bone marrow MSCs, enhanced the biomineralization of cells, as well as differentiation and resulted in a significant up-regulated bone marker gene expression of type I collagen, OCN, ALP and BSP. In addition to the positive effects of Zn^+2^ on osteogenic differentiation, studies have evaluated the EMF effect on osteogenic promotion and osteoporosis treatment [45]. The results obtained in the study of Kaya et al. (2011) [46] and Manjhi et al. (2013) [16] indicated that 50 Hz, 1.5 mT EMF and chronic (2 h/d × 8 wk) ELF-MF exposure (17.96 micro-Tesla, 50 Hz) to SCI rats for 6 months may be useful in the prevention and treatment of osteoporosis, respectively. In another study, it was shown that PEMF could increase ALP activity, OCN synthesis and collagen production [47]. The results of this study are in line with the results of a previous study, which showed that 50 Hz, 20 mT EMF increased ALP activity, which is an early marker of osteogenic differentiation in ADSCs [29]. In this study, it was shown that 0.432 μg/ml ZnSO_4_ and 50 Hz, 20 mT EMF promoted osteogenesis differentiation in MSCs.

Till date, the roles of cAMP/PKA, MAPK/ERK1/2 and Wnt/β-catenin signaling pathways in osteoblast differentiation, as well as various aspects of skeletal development and bone maintenance are still being disputed [20, 22, 24]. Siddappa et al. (2008) described the osteogenic differentiation effect of cAMP/PKA pathway in hMSCs [24]. Hu (2003) indicated that p38 MAPK was required for BMP2 induced differentiation in osteoblasts [48]. In another study, ERK1/2, as a member of the MAPK family, was also reported to be implicated in BMP2 and 1,25(OH)2D3– induced osteoblast differentiation [49]. In a study by Takada et al. (2009), the important role of Wnt signaling pathway in osteoblastogenesis was shown [50]. The effects of EMF in the activation of signaling pathways, such as PKA, MAPK and Wnt/β-catenin, have also been reported. Hogan and Wieraszko (2004) demonstrated that 0.16 Hz, 15mT EMF can regulate the concentration of cAMP [38]. In another study, Hou et al. (2006) showed that EMF could activate the MAPK signaling pathways of human retinal pigment epithelial cells [27]. More recently, Wang et al. (2014) indicated that Wnt/β-catenin signaling pathway might be involved in the synergistic induction of the osteogenic differentiation of amniotic epithelial cells by PEMF and osteogenic induction medium [51]. In this study, PKA, ERK1/2 and Wnt/β-catenin signaling pathways were detected to determine if 0.432 μg/ml ZnSO_4_ and 50 Hz, 20 mT EMF could also activate these pathways.

The results of the present study showed that 0.432 μg/ml ZnSO_4_ and 50 Hz, 20 mT EMF activated these pathways through increase in ALP and PKA activities, calcium and cAMP levels and expression of ALP, OCN, Runx2 and BMP2 as osteogenic differentiation genes, as well as expression of β-catenin, Wnt1, Wnt3a and LRP5 as Wnt/β-catenin pathway-related genes. In a study by Ishido et al. (2001), it was demonstrated that 50 Hz at 1.2 μT, as well as 100 μT magnetic field resulted in an increase in cAMP level [52]. While Yuge et al. (2003) showed that magnetic microparticles accelerated the osteoblast differentiation via activation of p38 phosphorylation (MAPK), but not p-ERK1/2 [23]. It seems that the use of magnetic fields with different frequencies and intensity to stimulate osteogenesis represent a considerable challenge. The present investigation, therefore, focused on the differentiation of osteoblast induced by 50 Hz, 20 mT EMF and the accompanying effects of Zn^2+^. To further demonstrate the correlation between the signaling pathways and the osteogenic markers induced by the magnetic field in the presence of Zn^+2^, the inhibitors of PKA, and ERK1/2 pathways were used to determine whether inhibiting them could also reduce the osteogenic markers of the ADSCs induced by the magnetic fields and Zn^+2^. This study's results showed that the inhibition of the PKA and ERK1/2 signaling pathways reduced the expression of ADSCs osteogenic markers, such as ALP, OCN, Runx2 and BMP2. However, the findings of this study are consistent with the hypothesis that 50 Hz, 20 mT EMF, in the presence of 0.432 μg/ml ZnSO_4_, can promote ADSCs osteogenic differentiation via the cAMP–PKA and MAPK pathways. Although the underlying mechanism remains unclear, but few studies have been published in relation to the implicated mechanisms on osteogenesis, which can be effective in finding the fundamental mechanism. In a study, Yamaguchi et al. (1994) reported that the effect of ZnSO_4_ and zinc-chelating 13-alanyl-L-histidine (AHZ) on increase in ALP activity, protein, and DNA contents in osteoblastic MC3T3-EI cells was completely abolished by the presence of cycloheximide, an inhibitor in translational process of protein synthesis. Presumably, ZnSO_4_ and AHZ can stimulate protein synthesis in osteoblastic cells. In fact, this experiment showed that ZnSO_4_ and AHZ can stimulate protein synthesis in the translational process of osteoblastic MC3T3-EI cells via direct activation of aminoacyl-tRNA synthetase, a rate-limiting enzyme, which synthesizes aminoacyl-tRNA to initiate protein synthesis [12]. In another study, Li et al. (2011) investigated that Panax notoginseng saponins (PNS) could promote bone formation via stimulating the activation of ERK and p38 pathway by increased phosphorylation of these proteins [53]. According to the aforementioned reports, in the present study, it was suggested that the enhancement of osteogenic differentiation in the presence of 0.432 μg/ml ZnSO_4_ and 50 Hz, 20 mT EMF via PKA, ERK1/2 and Wnt/β-catenin signaling pathways, may be due to changes in osteoblastic gene expression, osteoblastic protein synthesis or/and phosphorylation of signaling pathway proteins.

In conclusion, this research indicated that a 50 Hz, 20 mT EMF, in the presence of 0.432 μg/ml ZnSO_4,_ promoted ADSCs osteogenesis differentiation, and that it correlated with the PKA, ERK1/2 and Wnt/β-catenin signaling pathways.
